# Risk of Stroke after Herpes Zoster – Evidence from a German Self-Controlled Case-Series Study

**DOI:** 10.1371/journal.pone.0166554

**Published:** 2016-11-23

**Authors:** Tania Schink, Sigrid Behr, Kathrin Thöne, Hélène Bricout, Edeltraut Garbe

**Affiliations:** 1 Leibniz Institute for Prevention Research and Epidemiology – BIPS, Bremen, Germany; 2 University Cancer Center Hamburg (UCCH), University Medical Center Hamburg-Eppendorf (UKE), Hamburg, Germany; 3 Epidemiology Department, Sanofi Pasteur MSD, Lyon, France; Kaohsiung Medical University Hospital, TAIWAN

## Abstract

**Background:**

Herpes zoster (HZ) is caused by reactivation of the latent varicella-zoster virus (VZV). A severe complication of HZ is VZV vasculopathy which can result in ischemic or hemorrhagic stroke. The aims of our study were to assess the risk of stroke after the onset of HZ and to investigate the roles of stroke subtype, HZ location and the time interval between HZ onset and stroke.

**Methods:**

A self-controlled case-series study was performed on a cohort of patients with incident stroke recorded in the German Pharmacoepidemiological Research Database (GePaRD), which covers about 20 million persons throughout Germany. We estimated adjusted incidence rate ratios (IRR) by comparing the rate of stroke in risk periods (i.e., periods following HZ) with the rate of stroke in control periods (i.e., periods without HZ) in the same individuals, controlling for both time-invariant and major potentially time-variant confounders.

**Results:**

The cohort included 124,462 stroke patients, of whom 6,035 (5%) had at least one HZ diagnosis identified in GePaRD either as main hospital discharge diagnosis or as HZ treated with antivirals. The risk of stroke was about 1.3 times higher in the risk periods 3 months after HZ onset, than in the control periods (IRR: 1.29; 95% confidence interval: 1.16–1.44). An elevated risk of similar magnitude was observed for ischemic and unspecified stroke, but a 1.5-fold higher risk was observed for hemorrhagic stroke. A slightly stronger effect on the risk of stroke was also observed during the 3 months after HZ ophthalmicus (HZO) onset (1.59; 1.10–2.32). The risk was highest 3 and 4 weeks after HZ onset and decreased thereafter.

**Conclusions:**

Our study corroborates an increased risk of stroke after HZ, which is highest 3 to 4 weeks after HZ onset. The results suggest that the risk is more pronounced after HZO and is numerically higher for hemorrhagic than for ischemic stroke.

## Introduction

Herpes zoster (HZ) is caused by an endogenous reactivation of the latent varicella-zoster virus (VZV). Primary VZV infection usually occurs during childhood and results in varicella (chickenpox). After this, VZV persists asymptomatically in a latent state in the dorsal roots of the cranial and spinal ganglia. When VZV-specific cell-mediated immunity declines due to advancing age, immune-compromising diseases, or immunosuppressant or immuno-modulatory medication, VZV can reactivate resulting in HZ (shingles). In most cases, HZ presents with a characteristic, painful unilateral dermatomal vesicular rash affecting one or two dermatomes.

A severe complication of HZ is VZV vasculopathy which can result in ischemic or hemorrhagic stroke.[1] Several epidemiological studies from Taiwan, Sweden, Denmark, the UK, and the US have investigated the risk of stroke following HZ over different time intervals.[2–9] Two cohort studies from Taiwan and Sweden observed a 1.3 fold increased risk of any (ischemic or hemorrhagic) stroke within 12 months after HZ.[3, 8] Another cohort study based on the Danish national registers in adults reported a 2.3-fold increased risk in the first two weeks, a 1.2-fold increased risk between two weeks and one year and a 1.1-fold increased risk after the first year following HZ as compared to the baseline period.[7] A similar temporal pattern was observed by Langan et al. in a study based on the UK Clinical Practice Research Datalink (CPRD) database in adults: the risk of stroke was 1.6-fold increased in the first month, 1.4-fold increased between one and three months, 1.2-fold increased between four and six months and not increased between seven and twelve months after HZ.[4] A similar temporal trend was also observed in a recently published US community cohort study (1986-2011) where Yawn et al. found a 1.5-fold increased risk of stroke in the first 3 months after HZ among patients aged 50+ which declined beyond 3 months.[9] In another US study based on Medicare beneficiaries older than 65 years, the observed risks for ischemic stroke were highest in the first week after HZ (2.7-fold increase) and decreased to baseline risk after 3 months.[6] Breuer et al. examined also the long term effects of HZ on stroke risks in a matched cohort study based on data from the UK THIN database and found no increased risk of stroke after a median follow-up of 6.3 years.[2] The risk of stroke associated with HZ ophthalmicus (HZO) was found to be higher in studies that differentiated by HZ site.[3–6]

Six of these studies suffered from several limitations: the Swedish and Taiwanese cohort studies were of small sample size (111 stroke patients with HZ in Sweden, 133 and 53 stroke patients with HZ and HZO, respectively, in Taiwan) and measured the risk during only one time interval, i.e. after one year.[3, 5, 8] The Danish cohort study identified HZ patients based on acyclovir prescriptions which could give rise to misclassification of herpes simplex virus infection as HZ infections.[7] All six studies had the potential for residual confounding [2, 3, 5, 7–9] due to missing information on important risk factors of stroke. The studies by Langan et al. and Minassian et al. were not subject to these limitations since they included large numbers of stroke patients with recorded HZ (6,500 and 42,900, respectively) and used the self-controlled case-series (SCCS) study design which controls by design for time-invariant confounders.[4, 6] Except for age, both studies did not control for time variant confounders over a median follow-up period of 12.5 and 5 years, respectively. It is likely that the prevalence of some major risk factors such as atrial fibrillation and flutter, transient ischemic attacks and myocardial infarctions as well as the use of antithrombotic medication and statins changed during this time span.

The aims of our study were to assess the adjusted risk of stroke after HZ onset based on a large German population database and in particular the assessment of the specific risks of ischemic and hemorrhagic stroke, of the risk of stroke after HZO, and of the temporal pattern of the HZ-related risk and to examine the effect of controlling for both time-invariant and major potentially time-variant confounders.

## Methods

### Data source

Data was obtained from the German Pharmacoepidemiological Research Database (GePaRD) which is based on claims data from four statutory health insurance providers covering over 20 million persons throughout Germany. GePaRD contains individual level information on demographic characteristics, hospitalizations (including admission diagnoses, main discharge diagnoses, and reason for discharge including death), outpatient physician visits, and outpatient drug dispensation for reimbursed products. While exact dates are provided for hospitalizations and outpatient drug dispensations, only the year and quarter are known for outpatient diagnoses. The acceptability of GePaRD for pharmacoepidemiological research has been assessed methodologically as well as by validation studies.[10–15] Recently, GePaRD has been used for various types of pharmacoepidemiological studies including drug utilization studies and studies investigating the risks of drugs or vaccines [10, 16–18].

In Germany, the utilization of health insurance data for scientific research is regulated by the Code of Social Law. All involved health insurance providers as well as the Senator for Science, Health, and Consumer Protection in Bremen and the German Federal (Social) Insurance Office as their responsible authorities approved the use of the data for this study. Informed consent was not required by law, since the study was based on pseudonymous data.

### Study design

The SCCS design [19] was used to assess the risk of stroke after HZ by comparing the incidence of stroke in "risk periods" after an HZ episode with the incidence of stroke during "control periods" without HZ within the same individual. By implicitly controlling for time-invariant variables this design reduces residual confounding.

### Study Population

For this study, data of 7.7 million persons from 2004 to 2011 was used, since at the time of analysis no more recent data was available. Patients of all ages were included into the study cohort if they (i) had been insured for at least 12 consecutive months between January, 2004 and December, 2011, (ii) had a diagnosis of ischemic, hemorrhagic, or unspecified stroke during the observation period, i.e. after cohort entry, and (iii) had no diagnosis of HZ or stroke within the 12 months preceding cohort entry (baseline period). Patients with HZ during follow-up were required to have an exact date of HZ onset which could only be retrieved from HZ-related admissions to hospital or from prescriptions of HZ related antiviral medication. Hence, patients whose first HZ diagnosis during follow-up was an outpatient diagnosis without accompanying prescription of systemic antiviral treatment (acyclovir, brivudin, famciclovir, valaciclovir) were excluded.

Patients without HZ during the observation period were also included in this study. These patients did not directly impact the estimated risk of stroke in time periods following HZ but contributed to efficient estimation of the effects of other variables included in the multivariable analysis such as age and vascular risk factors.

### Outcome definition

Cases of ischemic, hemorrhagic, and unspecified stroke were identified by searching in the main discharge diagnoses of hospitalizations for the ICD-10 GM codes (German Modification of the International Classification of Diseases) I61.-, I63.- and I64.-, respectively. The date of stroke was defined as the corresponding admission date. Only the first diagnosis of stroke was considered as the occurrence of subsequent strokes is influenced by previous events, and the SCCS design requires recurrent events to be independent of each other.[19]

### Identification of HZ

ICD-10 GM diagnostic codes for HZ (B02.-) were used to identify HZ cases from the physician visits and hospitalization data. HZO was identified by the specific ICD-10 GM diagnostic code B02.3. For hospitalization data, the main hospital discharge diagnosis was used and the date of zoster onset was defined as the admission date. For outpatient data, diagnoses of HZ qualified as "certain" or "suspected" by the respective physician were both considered. Date of onset for these cases was defined as the date of systemic antiviral (acyclovir, brivudin, famciclovir, valaciclovir) prescription. Recurrent episodes of HZ were also considered, and were defined as a HZ episode after at least three months free of HZ.

### Definition of risk periods and time-varying variables

**Fig 1:**
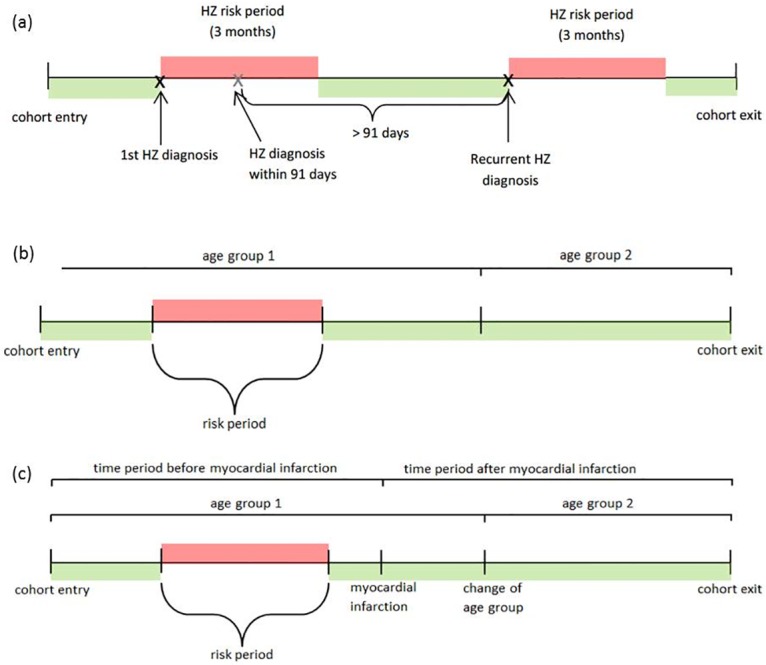
Definition of risk periods: (a) for primary analysis; (b) split by age group; (c) additional stratification by potential confounders (here: myocardial infarction).

The observation time of each individual was divided into "risk periods" and "control periods". Risk periods denote the periods after a HZ onset, during which an individual was considered to have an elevated risk of stroke. For the primary analysis, the beginning of the risk period was defined as the date of HZ onset, and the end of the risk period as 91 days thereafter (Fig 1a). In a secondary analysis the end of the risk period was further divided into intervals of 1 to 14 days, 14 days to 1 month, 2 to 3 months, 4 to 6 months, and 7 to 12 months after HZ onset. Any period of follow-up that was more than 12 months, either before or after HZ onset, was considered as a control period. To account for changes in age over time, the observation period was also split into 5-year-age periods (Fig 1b). Separate analyses were performed to control for potential time-varying confounders (see below), in which the follow-up time was additionally stratified by the respective potential confounder (Fig 1c).

### Statistical Analysis

In the primary analysis, a log-linear Poisson model was fitted to estimate the occurrence of the combined endpoint of ischemic, hemorrhagic, and unspecified stroke, taking into account the time-dependent effects of age and HZ, using a risk period of 3 months after HZ onset. Secondary analyses included subdivision of the risk period, stratification by stroke subtype (hemorrhagic vs. ischemic/unspecified stroke), and investigation of the effect of HZO.

Sensitivity analyses were performed to evaluate the robustness of the primary analysis with respect to the violation of assumptions of the SCCS study design. To rule out any impact of strokes with fatal outcome, the primary analysis was repeated after exclusion of patients with fatal stroke. Effects of potentially time-varying vascular risk factors (myocardial infarction, transient cerebral ischemic attack, atrial fibrillation and flutter identified from inpatient and outpatient diagnoses; use of antithrombotics, use of statins identified from outpatient dispensations) were investigated in separate analyses. The first occurrence of the respective codes in the baseline period or during follow-up was used to define the status of the time-dependent covariable. To assess the change in prevalence of these risk factors over time, their prevalence during the baseline period as well as in the last year of the observation period was described. Codes for the vascular risk factors are available upon request.

All statistical analyses were conducted using SAS version 9.2 and 9.3. Since the SCCS method has not yet been implemented in standard statistical software, a published macro based on the SAS-IML programming language has been used for the SCCS analysis.[20]

## Results

The cohort included 124,462 patients; 81% had an ischemic stroke, 12% had a hemorrhagic stroke and 7% had an unspecified stroke. Nine percent of the patients died in hospital after being admitted for stroke. The average length of follow-up was 5.6 years (standard deviation 1.9). The majority of patients were female (58%) and the mean age of all patients was 71.3 years (standard deviation 12.5). Overall, 94% of cohort members were over 50 years of age (Table 1).

**Table 1:** Baseline characteristics of study cohort.

Characteristic	Cohort, N = 124,462	Cohort members with HZ, N = 6,035
Male sex, n (%)	52,135 (41.9)	2,103 (34.9)
Age, mean (Std)	71.3 (12.5)	72.6 (10.6)
Age categories, n (%)		
<30 years	743 (0.6)	14 (0.2)
30-<40 years	1,413 (1.1)	26 (0.4)
40-<50 years	5,289 (4.2)	145 (2.4)
50-<60 years	12,259 (9.8)	446 (7.4)
60-<70 years	29,627 (23.8)	1,483 (24.5)
70-<80 years	39,185 (31.5)	2,208 (36.6)
≥80 years	35.946 (28.9)	1,713 (28.4)
*Comorbidity*^a^, *n(%)*		
Atrial fibrillation and flutter	13,507 (10.9)	672 (11.1)
Myocardial infarction	5,621 (4.5)	272 (4.5)
Transient cerebral ischemic attack	6,864 (5.5)	368 (6.1)
*Comedication*^a^, *n (%)*		
Use of antithrombotics	33,424 (26.9)	1,687 (28.0)
Use of statins	22,251 (17.9)	1,227 (20.3)

HZ: herpes zoster; Std: Standard deviation

^a^Co-morbidity and co-medication were assessed in the 12 month prior to study inclusion.

During the observation time, 6,035 patients (5%) had at least one HZ diagnosis. Of these, 432 (7%) were diagnosed with HZO and 230 (4%) suffered from recurrent HZ. The 3-month risk periods following the first or recurrent HZ diagnosis comprised 1,560 person-years (0.2%) and the control periods encompassed 701,090 person-years. Of the strokes, 352 (0.3%) occurred during the 3-month risk periods and 124,112 during the control periods.

During the 3 months after HZ onset the risk of stroke was about 1.3 times higher than in the control periods (age-adjusted incidence rate ratio (IRR): 1.29, 95% CI: 1.16–1.44). An elevated risk of similar magnitude was observed for ischemic and unspecified stroke, whereas a 1.5 times higher risk was observed for hemorrhagic stroke (Table 2). A slightly stronger effect on the risk of stroke was observed during the 3 months after HZO onset (age-adjusted IRR: 1.59, 95% CI: 1.10–2.32).

**Table 2:** Risk of stroke after HZ and HZO compared to control periods.

	All HZ		HZO	
	No. of strokes in risk period	IRR (95% CI)	No. of strokes in risk period	IRR (95% CI)
**Risk period 3 months**				
Stroke (any type)	352	1.29 (1.16–1.44)	31	1.59 (1.10–2.32)
Ischemic and unspecified stroke	310	1.27 (1.13–1.42)	27	1.57 (1.05–2.35)
Hemorrhagic stroke	42	1.53 (1.11–2.11)	4	1.82 (0.62–5.37)
**Subdivided risk periods (secondary analysis)**				
<2 weeks	59	1.30 (1.00–1.68)	2	0.63 (0.16–2.53)
week 3–4	73	1.52 (1.20–1.91)	12	3.56 (1.99–6.38)
month 2–3	219	1.24 (1.08–1.42)	17	1.37 (0.84–2.25)
month 4–6	274	1.09 (0.97–1.24)	25	1.44 (0.96–2.17)
month 7–12	444	0.96 (0.87–1.06)	29	0.63 (0.87–1.35)

HZ: herpes zoster HZO: herpes zoster ophthalmicus IRR: incidence rate ratio CI: confidence interval

**Fig 2:**
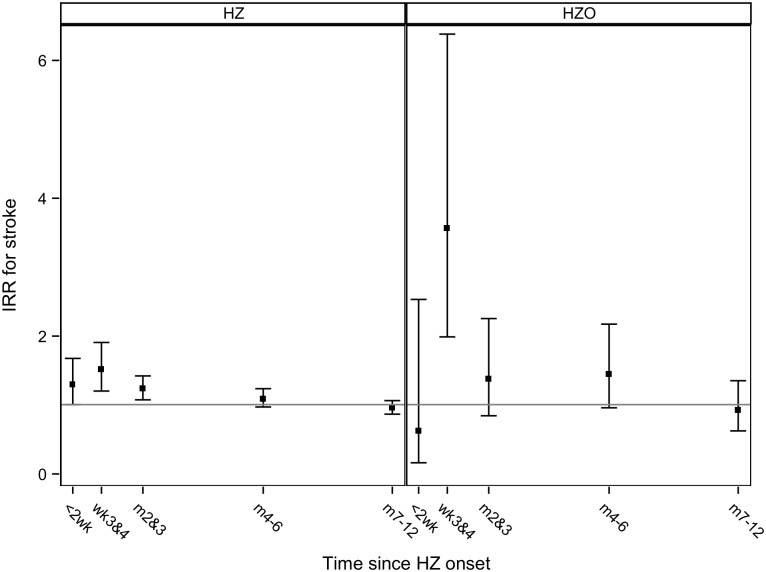
Incidence rate ratios (IRRs) with 95% confidence intervals for stroke after HZ as well as for stroke after HZO in time intervals < 2 weeks, week 3–4, month 2–3, month 4–6, and month 7–12.

Further subdivision of risk periods in secondary analyses revealed that the risk of stroke was highest 3 to 4 weeks after HZ onset, after which it decreased (Fig 2 and Table 2). Six months after HZ onset, no statistically significant difference was seen in the risk of stroke between the risk and control periods. The risk of HZO-associated stroke was also highest 3 to 4 weeks after onset (age-adjusted IRR: 3.56, 95% CI: 1.99–6.38), but was numerically higher than that for all HZ.

Sensitivity analysis on the impact of fatal stroke and potential time-varying confounders confirmed the results of the primary analysis (Table 3). Although the occurrence of many vascular risk factors considerably changed over time (Table 4), inclusion of these time-varying variables did not change the effects of HZ on the risk of stroke (Table 3).

**Table 3:** Sensitivity analyses: exclusion of fatal stroke and adjustment for time-varying confounders.

	No. of strokes in risk period	IRR (95% CI)
**Exclusion of fatal stroke (N = 112,719)**	319	1.29 (1.15–1.44)
**Adjustment for time-varying confounder**		
Myocardial infarction	352	1.29 (1.16–1.44)
Transient cerebral ischemic attack	352	1.29 (1.15–1.43)
Atrial fibrillation and flutter	352	1.28 (1.15–1.43)
Antithrombotic medications	352	1.32 (1.18–1.47)
Statins	352	1.31 (1.18–1.46)

IRR: incidence rate ratio CI: confidence interval

**Table 4:** Distribution of time-varying confounders.

Variable	Baseline period	Observation period^a^
Myocardial infarction	5,621 (4.5%)	11,777 (9.5%)
Transient cerebral ischemic attack	6,864 (5.5%)	18,122 (14.6%)
Atrial fibrillation and flutter	13,507 (10.9%)	41,132 (33.0%)
Anticoagulants	11,815 (9.5%)	25,116 (20.2%)
Platelet aggregation inhibitors excl. heparin and ASA	6,533 (5.2%)	20,974 (16.9%)
Other Antithrombotics (enzymes)	14 (<0.1%)	132 (0.1%)
ASA (used as platelet aggregation inhibitors)	16,611 (13.3%)	28,682 (23.0%)
ASA (used as analgesics)	2,722 (2.2%)	5,269 (4.2%)
Statins	22,251 (17.9%)	55,839 (44.9%)

^a^ Only the last year of the observation period was considered.

## Discussion

To our knowledge this is the first study examining the association between HZ and stroke, as well as stroke subtypes (hemorrhagic, ischemic and unspecified stroke), controlling for both time-invariant and major potentially time-variant confounders such as atrial fibrillation and flutter, transient ischemic attack and myocardial infarction as well as the use of antithrombotic medication and statins.

In this large retrospective cohort study using the SCCS design, we observed a 1.3-fold increase in the risk of stroke (any type) within 3 months of HZ onset. Sensitivity analyses demonstrated that this result was robust with respect to time-varying confounders despite observed changes in the prevalence of the investigated vascular risk factors over the observation period. The risk of stroke was 1.5-times higher 3 to 4 weeks after HZ onset, and decreased thereafter. At 6 months after HZ onset the risk was no longer statistically significantly elevated. Risks of stroke were even more pronounced following HZO. With respect to stroke subtypes, higher rates of hemorrhagic stroke were observed than for ischemic and unspecific stroke following HZ or HZO.

The temporal pattern of the risk of stroke was similar to those observed by Langan et al., Minassian et al., and Sreenivasan et al.[4, 6, 7] In all three studies, the risk of stroke was higher in the first weeks following HZ and returned to baseline risk after 1 year. The peak observed by Sreenivasan et al. and Minassian et al. was within the first week after HZ onset, compared to 2 to 4 weeks in our study, and 1 to 2 months for patients treated with antivirals in Langan et al. This difference might be explained by the different definitions of HZ onset used in these studies. Sreenivasan et al defined HZ onset as the start of antiviral therapy; Minassian et al. used start of antiviral therapy within 7 days before or after HZ diagnosis. In contrast, we defined HZ onset as admission to hospital or start of antiviral therapy and Langan et al. simply assessed the period after HZ diagnosis. The risk of stroke after HZ and HZO observed in our study was well comparable with the risk reported by Langan et al. for patients treated with antivirals. The 3.6-fold risk (95% CI: 2.0–6.4) observed between 3 to 4 weeks after HZO onset was in line with the risks reported in the two Taiwanese studies (4.5- and 4.3-fold increased risk within 1 year following HZO).[3, 5]

Our study suggests a slightly higher risk of hemorrhagic strokes than ischemic and unspecified strokes following HZ onset. This is consistent with the Taiwanese study by Kang et al., which observed an even more pronounced risk of hemorrhagic stroke (2.8-fold risk compared to 1.5-fold risk in this study). This higher risk might be explained by ethnic differences, as it has been indicated in the literature that Asian ethnicity is associated with higher risks of hemorrhagic stroke.[21] The risk of hemorrhagic stroke found by Minassian et al. was comparable to ours. However, this risk was lower than the risk observed for ischemic stroke in the other study from the US which might be explained by the older age (median 80) of the population included in the study by Minassian.

A major strength of our study is the size of the study sample, with more than 120,000 stroke patients of whom 6,035 had HZ during the study period and the high percentage of specific coding of strokes (93%), which allowed us to calculate separate risk estimates for ischemic and hemorrhagic stroke. In the study by Langan et al. based on 6,584 patients with incident stroke and HZ, about 60% of strokes were coded as unspecified and only 6% (n = 422) were coded as hemorrhagic which precluded any conclusions for stroke subtype specific risks.

Most previous studies could not adequately assess important risk factors such as life-style factors, socio-economic status and family history of stroke. By using the SCCS design these probably time-invariant confounders were implicitly controlled for in our study. However, given the average follow-up time of 5.6 years, the prevalence of major risk factors e.g., atrial fibrillation and flutter, transient ischemic attack, and myocardial infarction probably changed, as likely did the use of antithrombotic medication and statins, as was already seen in our study with shorter follow-up (Table 4). Additionally, age is a potential confounder as both risk of stroke and incidence of HZ increase with age. Therefore we also adjusted for these time-varying confounders by stratifying the observation period accordingly and thus conducted the—to our knowledge—first study adjusting both for normally unmeasurable time-invariant and important time-variant risk factors.

Although herpes simplex cases may have been misclassified as HZ we consider the risk of misclassification of HZ was minimized in our study as the clinical picture of HZ is quite characteristic and can be reliably diagnosed. Moreover, we excluded patients with an outpatient diagnosis of HZ who received no antiviral therapy which has been shown to improve the positive predictive value.[22] In a previous study conducted by our group, 70% of HZ patients received systemic antiviral medication.[23] Although treatment percentages were comparable between immuno-compromised and immuno-competent patients as well as between patients with complicated and uncomplicated HZ, we cannot exclude that the risk of stroke might differ in HZ patients treated and untreated with systemic antivirals, which has been reported by Langan et al. Regarding the anatomical location of HZ, the data only allowed for identification of HZO which was defined based on diagnosis codes. With this approach based on diagnosis codes alone we may have missed some cases of HZO, however, we consider it to be very specific for HZO leading to a high positive predictive value.

Misclassification of stroke is also possible, but previous studies in other databases have shown that ICD-10 codes of stroke usually have good positive predictive value.[24]

For valid estimation of the risks, the SCCS design requires that the occurrence of exposures (i.e. HZ) be independent of previous events (i.e. strokes) and that the observation period not be censored or affected by an event.[19] Both of these assumptions are violated when a stroke results in death. Thus we conducted sensitivity analyses excluding patients with a fatal stroke to evaluate the impact of fatal strokes, as recommended by Whitaker et al.[19] However their exclusion did not change the estimate of the IRRs nor the corresponding CIs, suggesting that the potential violation of the independence assumption had no impact on the results.

The mechanism underlying the observed risk of stroke following HZ is likely to be multifactorial, including VZV vasculopathy due to cerebral arterial infection and systemic inflammation observed after acute reactivation of latent VZV infection.[1, 25] The true incidence of VZV vasculopathy is not known, especially as it has been described that VZV can reactivate in the absence of rash.[26]

In summary, our study corroborates an increased risk of stroke after HZ after controlling for time invariant and time-varying confounders. The magnitude of the risk was up to a 50% increase 3 to 4 weeks after HZ onset. In line with the trends observed in previous studies, the results suggest that the risk is more pronounced after HZO and is numerically higher for hemorrhagic than for ischemic stroke.
